# Detection of miRNA as Non-Invasive Biomarkers of Colorectal Cancer

**DOI:** 10.3390/ijms16022810

**Published:** 2015-01-27

**Authors:** Albert Ren, Yujuan Dong, Ho Tsoi, Jun Yu

**Affiliations:** 1 Institute of Digestive Disease and Department of Medicine and Therapeutics, State Key Laboratory of Digestive Disease, Li Ka Shing Institute of Health Sciences, CUHK Shenzhen Research Institute, Prince of Wales Hospital, The Chinese University of Hong Kong, Shatin, New Territories, Hong Kong, China; E-Mails: albertren@u.northwestern.edu (A.R.); yujuan_dong@yahoo.com (Y.D.); frankie_tsoiho@yahoo.com.hk (H.T.); 2 Weinberg College of Arts and Sciences, Northwestern University, 633 Clark Street, Evanston, IL 60208, USA; 3 Division of Colorectal Surgery, Department of Surgery, The Chinese University of Hong Kong, Shatin, New Territories, Hong Kong, China

**Keywords:** colorectal cancer, miRNA, circulatory, fecal, biomarker, diagnostic

## Abstract

Colorectal Cancer (CRC) is one of the deadliest cancers—ranking as the fourth most common cause of cancer-related deaths in the world. It is such a deadly disease because it is largely asymptomatic until the latter stages—oftentimes when the cancer has metastasized. Thus, a huge emphasis of cancer treatment is placed on early detection. Currently, there is a lack of a noninvasive, reliable, and cost-effective screening method for CRC. In recent years, microRNA (miRNA) diagnostic markers have been suggested as a viable new screening method for CRC. miRNAs play an important role in carcinogenesis, and has been observed to be dysregulated in many cancers including CRC. This review examines the diagnostic potential of circulatory and fecal miRNA markers in relation to CRC, as well as current techniques to detect them.

## 1. Introduction

Colorectal cancer (CRC) is one of the deadliest killers in the world, ranking as the fourth most common cause of cancer-related deaths [1]. Every year, there are more than 1.2 million new cases and over 600,000 deaths worldwide [2]. While CRC mortality rates have declined in Western societies due to increased endoscopic screening, CRC continues be an increasingly prevalent problem in Asia [2]. The risk factors for CRC are well known such as age, sex, family history, pre-existing conditions (inflammatory bowel disease, lynch syndrome,* etc.*) and other preventable lifestyle factors (low fiber diet, diet high in red meat, physical inactivity, obesity, smoking, alcohol) [3]; however, CRC continues to be such a serious health problem because it is largely asymptomatic until the latter stages—oftentimes when the cancer has already metastasized. If CRC is detected early, or as an adenomatous polyps, it is a largely treatable disease that can benefit from curative surgery [4].

Currently, there is a lack of a low-cost, noninvasive screening method that has high sensitivity and specificity. The most commonly used screening tests for CRC are colonoscopies, flexible sigmoidoscopies, guaiac-based fecal occult blood tests (FOBT), and fecal immunochemical tests (FIT) [5]. While colonoscopies are highly reliable for detecting CRC, they are invasive, costly, and present a small but significant risk for perforations [6]. Therefore, these factors have hindered patient compliance and a worldwide application of this procedure [7]. Flexible sigmoidoscopies do not require sedation and is a more simple procedure to perform but provides low diagnostic sensitivity and specificity for cancers in the proximal colon [6]. Because proximal advanced adenomas can often exist in the absence of distal lesions, flexible sigmoidoscopies may produce misleading test results and give patients a false sense of security [6]. FOBTs are noninvasive and inexpensive to administer but also suffer from low diagnostic sensitivity and specificity [8]. The test relies on the detection of peroxidase in haeme caused by cancer bleeding but test results can oftentimes be confounded by factors such as the high consumption of red meat or other peroxidase-containing foods [6]. FITs are similar to FOBTs but rely on human antibodies to detect cancer bleeding in the lower digestive tract. Because FITs have higher sensitivities and specificities than FOBTs, the medical community in recent years has recommended the switch from FOBTs to FITs as the primary form of noninvasive diagnostic test for CRC [2]. However, the antibodies used in FITs may be unstable so complications may arise during transport or storage [9].

Other less popular screening methods include CT colonography, capsule endoscopy, barium enema, and stool DNA testing but these tests also suffer from similar problems.

In recent years, the medical community has investigated the potential of using microRNA (miRNA) as a novel biomarker for the detection of CRC. miRNAs are a class of small, non-coding RNA molecules that regulate gene expression by inducing mRNA degradation or suppressing translation [10]. Because miRNAs have been identified as playing an important role in the aberrant gene expression of cancer cells and appear to be cell-type and disease specific, [11] they seem to be good candidates as diagnostic biomarkers. These biomarkers can be identified and measured via noninvasive methods in stool and blood samples, and show promise in the development of an effective and more cost-efficient screening test for CRC.

## 2. Current Techniques to Detect miRNAs in a Clinical Setting

Many studies have now successfully investigated and reported the potential of miRNA as diagnostic biomarkers. However, the road to producing a highly efficient, robust, and standardized method for miRNA detection in clinical practices still remains a long one. Herein, we summarize the issues and current state of research regarding miRNA detection.

In order for miRNA to be used as a diagnostic biomarker, it must first be isolated from either blood or stool samples. The techniques used for both sources are quite similar, and typically require the use of a commercial kit using acid guanidinium thiocyanate phenol-chloroform extraction. While RNA extraction is a fairly simple and straightforward technique, there are a few important technical issues that must be considered before miRNA biomarkers can be adopted for clinical use.

First, circulatory miRNAs in blood can be extracted from both serum and plasma, but recent studies have recommended plasma to be a more suitable source for miRNA extraction [12]. This is primarily due to the fact that the coagulation process in serum can cause hemolysis, which causes additional RNA to be released and can lead to artificially higher miRNA levels in samples [12]. For instance, Wang* et al.* [12] reported miRNA levels to be significantly higher in serum than in plasma for samples obtained from the same individuals.

Secondly, differences in plasma preparation can also alter circulatory miRNA levels which may affect the consistency and reliability of miRNA biomarkers. Cheng* et al.* [13] found that variations in sample processing—such as varying the amount of filtration and centrifugation—affected the levels of residual platelets in samples, which in turn, significantly affected the levels of miRNAs detected. Contamination of the plasma with cells from the supernatant, as well as variations in the choice of anti-coagulant used can also affect miRNA measurements [14]. Thus, quality control mechanisms such as additional centrifugation, the rejection of samples with platelet counts above a certain threshold, and the measurement of hemolysis are recommended in order to control for biases that might confound the measurement of miRNA levels [13].

Third, several studies have found variations in the amount and quality of RNA extracted based on the various kits used. Monleau* et al.* [15] performed a cross-comparison of three commercial kits (Qiagen’s miRNeasy mini kit, Norgen’s Total RNA Purification Kit, and Macherey-Nagel’s NucleoSpin miRNAs Plasma kit), and discovered that the Qiagen kit produced the greatest miRNA yield from plasma. Similarly, Kroh* et al.* [14] evaluated the performance of two extraction kits manufactured by Qiagen and Ambion, and also discovered Qiagen to produce greater miRNA yield. In a separate study comparing Qiagen’s miRNeasy kit, Norgen’s Total RNA Purification Kit, and Ambion’s miRVana Kit, Li* et al.* [16] determined the RNA isolated using the Norgen kit and Qiagen kit to be better quality than that from the Ambion Kit (measured by % of miRNA in small RNA extracted). Thus, it is important to standardize extraction procedures to control for differences mRNA yield that might arise from different sample preparation rather than having a cancer-based origin.

Once miRNA is extracted from patient stool or blood samples, it can be quantified for subsequent analysis. While many techniques such as northern blot,* in situ* hybridization, microarray, and next-generation sequencing (NGS) have been used to detect and profile miRNAs, these techniques are not cost-efficient in a clinical setting. Instead, the easiest and most commonly performed technique to quantify miRNA is quantitative real-time PCR (qPCR). qPCR is a well-established procedure in which RNA is reverse transcribed to cDNA, then amplified and quantified in real-time [17]. The amplified product is labeled using a fluorescent marker, and the amount of fluorescence can be used to quantify the original transcript. The two most commonly used for miRNA detection are TaqMan probes and SYBR Green I [18]. In recent years, TaqMan probes have been the method of choice due to its higher specificity. This is due to the fact that SYBR Green binds to all double-stranded DNA and cannot discriminate between PCR products, causing it to potentially bind to nonspecific double-stranded DNA sequences [18]. Taqman probes, on the other hand, are designed to hybridize to a specific stretch of the amplified product [18].

There are many technical issues that must be considered when using qPCR-based profiling of miRNA. Once miRNA is extracted, the initial process of cDNA synthesis can introduce technical noise and cause up to 100-fold variations in RT yields [19,20]. Some of the technical noise is caused by differences in priming techniques. There are primarily two priming techniques used in commercial kits: one uses two unique primers, while the other uses a unique forward primer and a universal reverse primer. The second method using a universal reverse primer has been shown to exhibit less specificity [17]. Furthermore, certain primers and probes can exhibit preferential binding to particular miRNA sequences, which would cause bias during the amplification stage [21].

More importantly, there is currently a lack of consensus on an appropriate reference gene to quantify miRNA. This remains the biggest obstacle to the clinical utility of miRNA because almost all published studies have used a relative quantification method such as qRT-PCR to detect miRNA expression. In the past, many groups have used miR-16, RNU6B snRNA, SNORD43 snRNA or synthetic versions of *C. elegans* miRNAs such as cel-miR-39, cel-miR-54, and cel-miR-238 as control genes [4]. However, all of these reference genes have been met with a wide spectrum of opinion. For instance, miR-16 may be unsuitable since its expression levels can be affected significantly by hemolysis [22,23]. The spike-in *C. elegans* synthetic miRNAs are often used to normalize for technical variability during extraction rather than for biological variability, and thus cannot be used as endogenous controls [14]. A study evaluating seven candidate reference genes (let-7a, miR-16, miR-93, miR-103, miR-192, miR-451, and RNU6B) concluded miR-16 and miR-93 to be good reference genes, but found RNU6B to be unsuitable due to its low expression levels in the serum of gastic cancer patients [24]. In recent years, some groups have suggested the use of an absolute quantification method such as digital PCR (dPCR) to quantify miRNA. While this technology is still in its infancy, it does not require the use of a reference gene and has higher sensitivity than conventional qPCR methods [25,26].

## 3. Current Plasma/Serum miRNA Biomarkers

While there are many blood-based biomarkers in existence such as carbohydrate antigen 19-9 (CA 19-9) and carcinoembryonic antigen (CEA), they suffer from low sensitivity, particularly for early stage CRC [27]. Recently, miRNA-based biomarkers have shown the most promise as a CRC-specific screening test due to its high sensitivity and specificity, and ease of use.

Several studies have successfully profiled and identified possible circulatory miRNA markers using the detection techniques mentioned previously. Unlike mRNA, miRNAs are surprisingly stable in blood despite the high activity of ribonuclease which can rapidly degrade RNA [28,29]. These miRNAs are protected from enzyme degradation because they are packed in exosomes or vesicles, or bound to proteins/lipoproteins [30,31,32,33]. In addition, less than 2 mL of blood is required for miRNA detection in plasma, and these blood samples can be transported stably in ice packs while preserving miRNA content [7]. These factors make circulatory miRNAs a promising new tool for the development of a noninvasive screening test.

Despite the abundance of miRNA molecules that have been implicated in connection with CRC, two miRNAs that have received considerable attention are miRNA-92a and miRNA-21. miR-92a is part of the miR-17-92 pre-cursor cluster which generates six miRNAs: miR-17, miR-18a, miR-19a, miR-20a, miR-19b, and miR-92a [34]. In various studies, miR-92a has been shown to serve as an oncogenic factor that targets the BCL-2 family of proteins which regulates apoptosis [34,35]. In a renowned study, Ng* et al.* [36] reported plasma miR-92a to have a sensitivity and specificity of 89% and 70% respectively in distinguishing CRC patients from healthy controls. Huang* et al.* [37] also reported similar results (sensitivity: 84, specificity: 71.4). While these results are promising, Schee* et al.* [38] found miR-92a levels to be low in a substantial number of CRC tissue samples, and concluded that there was no reliable association between miR-92a levels and CRC. In addition, numerous studies have found miR-92a expression to be associated with other diseases such as hepatocellular carcinoma, breast cancer, and even cardiovascular diseases, pointing to the low disease specificity of miR-92a [39,40,41]. Thus, the pathological role of miR-92a still remains to be elucidated before it can be used reliably in a clinical setting.

Similarly, miR-21 has also been studied extensively in regards to CRC. It has been found to downregulate tumor-suppressor genes and induce cell proliferation [42]. Countless studies have cited the diagnostic potential of miR-21, with one study reporting that plasma miR-21 can predict CRC incidence with 90% specificity and sensitivity [7,38,43]. However, a few studies have had trouble verifying these findings and reported miR-21 expression to vary widely among CRC patients [38,44]. Most recently, it was reported that miR-21 levels can be preserved stably in serum during storage, suggesting that it is still a promising biomarker [45].

Despite the high diagnostic potential of individual miRNAs, reliable screening tests will typically employ a panel of multiple miRNAs to serve as biomarkers. Various studies have been performed to assess the efficacy of such a screening method. Kanaan* et al.* [46] performed a study evaluating 380 plasma miRNAs, and found a panel of 8 miRNAs (miR-532-3p, miR-331, miR-195, miR-17, miR-142-3p, miR-15b, miR-532, and miR-652) that could reliably differentiate patients with adenomatous polyps from healthy controls, as well as a panel of 5 miRNAs (miR-331, miR-15b, miR-21, miR-142-3p, and miR-339-3p) that could reliably differentiate advanced CR adenoma from CRC patients. The ability for a miRNA panel to differentiate premalignant polyps from healthy controls shows that miRNA biomarkers may be particularly useful for early detection of CRC due to the test’s noninvasive and cost-effective nature.

Giraldez* et al.* [47] also reported similar results evaluating plasma miRNA panels. However, the cohort size was much larger (63 CRC patients, 60 patients with CR adenoma, and 73 controls) than the cohort sized used in Kanaan’s study (26 controls, 16 patients with CR adenoma, and 45 CRC patients) [46,47]. They discovered a panel of 6 miRNAs (miR-18a, miR-19a, miR-19b, miR-15b, miR-29a, and miR-335) to be significantly upregulated in CRC patients, and found miR-18a to be significantly upregulated in patients with advanced adenomas [47]. Most notably, however, they could differentiate with high sensitivity and specificity early stage CRC as accurately as advanced stage CRC [47].

Most recently, Wang* et al.* [48] evaluated miRNAs panels isolated from serum as opposed to plasma, and identified a panel of 6 miRNAs that could accurately predict CRC incidence (miR-21, let-7g, miR-31, miR-92a, miR-181b, and miR-203) with 93% sensitivity and 91% specificity, significantly higher than conventional blood-based biomarkers such as CEA and CA19-9. For the same samples, the sensitivities of CA 19-9 and CEA were only 35% and 23% respectively [48]. Thus, circulatory miRNAs appear to be promising biomarkers particularly for preventative care and the early detection of CRC including adenomas.

## 4. Current miRNA Biomarkers from Stool

Conventional stool-based screening methods such as FOBTs and FITs suffer from a myriad of problems. FOBTs suffer from low sensitivity and specificity, and have poor patient compliance, while FITs suffer from low sensitivity for early stage CRC [49].

As a result, miRNA-based stool tests have been suggested in recent years as a potentially more effective screening method, particularly for diagnosing early stage CRC and pre-neoplastic lesions. Although the environment in stool is much more hostile than that of blood, miRNAs have been shown to remain intact and stable for detection in stool because they are packaged in exosomes [8,50]. In addition, because stool samples can be easily transported and requires at most 1 g for miRNA detection, miRNA-based stool tests seem to offer more efficiency and convenience than standard screening methods [8].

miRNA-based stool tests offer a few important advantages. Because fecal matter comes into direct contact with the lumen of the colon and may include cells exfoliated from malignant colonocytes, it is speculated that the earliest detectable molecular changes caused by CRC are present in stool rather than blood [51]. Secondly, miRNA dysregulation has been implicated in many pathways involving oncogenes and tumor suppressing genes such as WNT pathway activation, EGFR signaling activation, TGFβ inactivation, APC and TP53 gene-inactivating mutations, KRAS and BRAF gene-activating mutations [52,53,54]. Dysregulation of these pathways are characteristic of early cancer development, and therefore, miRNA-based screening tests might be better for early diagnosis compared with other stool-based screening tests such as FOBT or FIT.

Although fecal miRNAs have not been studied as extensively as circulatory miRNAs, the expression level of many fecal miRNAs have been observed to be dysregulated in patients with CRC or advanced adenomas. For instance, our group has observed miR-135b to be able to differentiate between different stages of tumor growth [55]. It can detect the presence of adenomas, advanced adenomas, and CRC with 62%, 73%, and 78% sensitivities respectively [55]. In addition, our group has also discovered miR-18a and miR-221 to be significantly upregulated in patients with CRC (with sensitivities of 62% and 61% respectively) [56]. However, there was no significant upregulation in patients with adenomas and advanced adenomas for these miRNAs [56]. Thus, this suggests that more research must be conducted in order to find the ideal miRNAs to serve as biomarkers for diagnosing CRC and pre-neoplastic lesions.

miR-92a and miR-21—which have been studied extensively in plasma—have also been observed to have higher expression levels in the stool of CRC patients [57]. However, the sensitivities and specificities of these two miRNAs for detecting CRC incidence were significantly lower in stool than in plasma (Wu *et al.* [57] reported miR-92a to have a sensitivity of 71.6% and a specificity of 73.3% while miR-21 had a sensitivity of 55.7% and specificity of 73.3%). The miR-17-92 cluster has also been investigated in patient stool samples, and can predict CRC incidence with 69.5% sensitivity and 81.5% specificity [58]. When combined with miR-21 and miR-135, the panel of markers can detect CRC with increased sensitivity (74.1%) and roughly the same specificity (79.0%). Thus, though individual fecal miRNAs in general suffer from lower sensitivities than plasma miRNAs, a carefully selected panel of fecal miRNAs can still produce a reliable biomarker for CRC with high sensitivity.

Most recently, Ahmed* et al.* [59] performed a study presenting the proof-of-principle application of miRNA biomarkers in stool tests. They found 12 miRNAs (miR-7, miR-17, miR-20a, miR-21, miR-92a, miR-96, miR-106a, miR-134, miR-183, miR-196a, miR-199a-3p and miR214) to be upregulated in the stool of CRC patients, and 8 miRNAs (miR-9, miR-29b, miR-127-5p, miR-138, miR-143, miR-146a, miR-222 and miR-938) to be downregulated in the stool of CRC patients. Using these 20 miRNAs, they could differentiate not only CRC incidences from healthy controls but also different TNM stages with high sensitivity and specificity. In addition, they improved the standard RNA isolation techniques by placing extracted RNA samples in a commercially available chaotropic agent to improve RNA quality. This has been innovative and marks an important advancement since fragmented RNA results in poor amplification, and ultimately faulty quantification.

## 5. Future Directions

The clinical utility of miRNA as noninvasive biomarkers for CRC is still in its infancy (Table 1). In order for miRNA biomarkers to be applied in a clinical setting, additional large-scale evaluation of miRNA markers using large, independent patient cohorts must be performed. Although individual miRNA markers have shown promise in detecting both early stage CRC as well as advanced stage CRC, effective screening tests will likely employ a panel of miRNA markers to improve reliability and consistency. However, this will likely be more expensive than measuring single miRNAs, and will require further studies to ensure that the optimal panel is selected to maximize sensitivity and specificity [4].

Secondly, detection protocols such as extraction and quantification methods, as well as normalization techniques must be standardized. While extraction from both serum and plasma is a relatively straightforward process, differences in sample preparation may lead to hemolysis or higher concentrations of residuals platelets, which may in turn, contribute to artificially higher levels of miRNA levels in samples [13,14]. Thus, it is important that standard quality control mechanisms be implemented to control for variations in miRNA levels that might be due to differences in detection methods rather than a cancer-based origin. Furthermore, nearly all studies up until now have used qPCR as a quantitation method for measuring miRNA [4]. The use of qPCR relies on the use of a reference gene to serve as a control, and differences in the reference gene used across studies introduces another variable that might cause bias in the quantitation of miRNA. Therefore, dPCR is a promising new tool that might address this issue since it offers absolute quantitation of miRNA without the need for a standard curve.

**Table 1:** Circulatory and fecal miRNAs as potential diagnostic markers for Colorectal Cancer (CRC).

miRNA	Sensitivity (%)	Specificity (%)	Sample Size, *n*	Endogenous Controls	Findings	Reference
*Circulatory miRNAs*						
miR-92a	CRC: 89	CRC: 70	140 (90 CRC, 50 control)	RNU6B	Upregulated in CRC Plasma	[36]
miR-92a	CRC: 84, AA: 64.9	CRC: 71.2, AA: 81.4	196 (100 CRC, 37 advanced adenomas, 59 control)	miR-16	Upregulated in CRC Plasma	[37]
miR-92a	N/A	N/A	316 (193 CRC)	RNU44	No reliable association with CRC found	[38]
miR-21	CRC: 90	CRC: 90	50 (30 training set, 20 plasma set)	U6	Upregulated in CRC Plasma	[43]
miR-21	N/A	N/A	316 (193 CRC)	RNU44	Significantly upregulated in CRC	[38]
miR-21	N/A	N/A	20 (5 control, 5 CRC)	18S rRNA	Upregulated in CRC	[7]
miRNA panel: miR-532-3p, miR-331, miR-195, miR-17, miR-142-3p, miR-15b, miR-532, and miR-652	CRC: 91 ADN: 88	CRC: 57 ADN: 64	CRC: 45 ADN: 16 Control 26	U6	Upregulated in CRC Plasma, Could differentiate Polyps from Controls	[46]
miRNA panel: miR-21, let-7g, miR-31, miR-92a, miR-181b, and miR-203	CRC: 93	CRC: 91	Training Set: 60 (30 CRC, 30 control) Validation Set: 142 (83 CRC, 59 Control)	miR-16	Up-regulated serum miRNAs: (miR-21, let7g), Down-regulated serum miRNAs (miR-31, miR-181b, miR-92a, miR-203)	[48]
miRNA panel: (miR-18a, miR-19a, miR-19b, miR-15b, miR-29a, and miR-335)	CRC: 78.57 AA: 80	CRC: 79.25 AA: 80	196 (63 CRC, 60 advanced adenoma, 73 control)	miR-16	Upregulated plasma miRNAs (miR-18a, miR-19a, miR-19b, miR-15b, miR-29a, and miR-335), miR-18a could differentiate adenoma from control	[47]
*Fecal miRNAs*						
miR-92a	CRC: 71.6 ADN: 56.1	73.3	133 (59 CRC, 74 control)	Equal amount of total RNA	Upregulated in stool of CRC patients	[57]
miR-21	CRC: 55.7	73.3	133 (59 CRC, 74 control)	Equal amount of total RNA	Upregulated in stool of CRC patients	[57]
miR-17-92 cluster	CRC: 69.5	81.5	316 (197 CRC, 119 control)	U6 snRNA	Upregulated in stool of CRC patients	[58]
miRNA panel: miR-17-92 cluster (miR-17, miR-18a, miR-19a, miR-19b, miR-20a, and miR-92a), miR-21, and miR-135	CRC: 74.1	79	316 (197 CRC, 119 control)	U6 snRNA	Upregulated in stool of CRC patients	[58]
miR-135b	CRC: 78 AA: 73 ADN: 62	68	424 (110 ADN, 59 AA, 104 CRC, 109 control, 42 IBD)	Equal amount of total RNA	Upregulated in stool of CRC patients	[55]
miR-221	CRC: 62	74	595 (151 ADN, 48 AA, 198 CRC, 198 control)	Equal amount of total RNA	Upregulated in stool of CRC patients	[56]
miR-18a	CRC: 61	69	595 (151 ADN, 48 AA, 198 CRC, 198 control)	Equal amount of total RNA	Upregulated in stool of CRC patients	[56]
Proof of Principle: miR-7, miR-17, miR-20a, miR-21, miR-92a, miR-96, miR-106a, miR-134, miR-183, miR-196a, miR-199a-3p, miR214, miR-9, miR-29b, miR-127-5p, miR-138, miR-143, miR-146a, miR-222 and miR-938	N/A	N/A	60 (40 CRC, 20 control)	18s rRNA	Upregulated in stool of CRC patients: miR-7, miR-17, miR-20a, miR-21, miR-92a, miR-96, miR-106a, miR-134, miR-183, miR-196a, miR-199a-3p and miR214 Downregulated in stool of CRC patients: miR-9, miR-29b, miR-127-5p, miR-138, miR-143, miR-146a, miR-222 and miR-938	[59]

Abbreviation: AA, Advanced Adenoma; ADN, Adenoma; CRC, colorectal cancer; IBD, inflammatory bowel disease; N/A: Not Available.

## Abbreviation

CRC: Colorectal cancer
miRNA: microRNA
FOBT: guaiac-based fecal occult blood tests
FIT: fecal immunochemical tests
NGS: next-generation sequencing
qPCR: quantitative real-time PCR
dPCR: digital PCR
CA 19-9: carbohydrate antigen 19-9
CEA: carcinoembryonic antigen

## References

[1] Ferlay J., Soerjomataram I., Dikshit R., Eser S., Mathers C., Rebelo M., Parkin D.M., Forman D., Bray F. (2014). Cancer incidence and mortality worldwide: Sources, methods and major patterns in GLOBOCAN 2012. Int. J. Cancer.

[2] Sung J.J., Lau J.Y., Young G.P., Sano Y., Chiu H.M., Byeon J.S., Yeoh K.G., Goh K.L., Sollano J., Rerknimitr R. (2014). An updated Asia Pacific Consensus Recommendations on colorectal cancer. Gut.

[3] American Cancer Society. http://www.cancer.org/cancer/colonandrectumcancer/detailedguide/colorectal-cancer-risk-factors.

[4] Kawamura M., Toiyama Y., Tanaka K., Inoue Y., Mohri Y., Kusunoki M. (2014). Can circulating microRNAs become the test of choice for colorectal cancer?. Curr. Colorectal Cancer Rep..

[5] Sung J.J., Lau J.Y., Young G.P., Sano Y., Chiu H.M., Byeon J.S., Yeoh K.G., Goh K.L., Sollano J., Rerknimitr R. (2008). Asia Pacific consensus recommendations for colorectal cancer screening. Gut.

[6] Ng S.C., Wong S.H. (2013). Colorectal cancer screening in Asia. Br. Med. Bull..

[7] Ahmed F.E., Amed N.C., Vos P.W., Bonnerup C., Atkins J.N., Casey M., Nuovo G.J., Naziri W., Wiley J.E., Allison R.R. (2012). Diagnostic microRNA markers to screen for sporadic human colon cancer in blood. Cancer Genomics Proteomics.

[8] Dong Y., Wu W.K.K., Wu C.W., Sung J.J.Y., Yu J., Ng S.S.M. (2011). MicroRNA dysregulation in colorectal cancer: A clinical perspective. Br. J. Cancer.

[9] Kumaravel V., Hayden S.P., Hall G.S., Burke C. (2011). New fecal occult blood tests may improve adherence and mortality rates. Clevel. Clin. J. Med..

[10] Bartel D.P., Lee R., Feinbaum R. (2004). MicroRNAs: Genomics, biogenesis, mechanism, and function. Cell.

[11] Hrašovec S., Glava D. (2012). MicroRNAs as novel biomarkers in colorectal cancer. Front. Genet..

[12] Wang K., Yuan Y., Cho J., Mcclarty S., Baxter D., Galas D.J. (2012). Comparing the MicroRNA spectrum between serum and plasma. PLoS One.

[13] Cheng H.H., Yi H.S., Kim Y., Kroh E.M., Chien J.W., Eaton K.D., Goodman M.T., Tait J.F., Tewari M., Pritchard C.C. (2013). Plasma processing conditions substantially influence circulating microRNA biomarker levels. PLoS One.

[14] Kroh E.M., Parkin R.K., Mitchell P.S., Tewari M. (2010). Analysis of circulating microRNA biomarkers in plasma and serum using quantitative reverse transcription-PCR (qRT-PCR). Methods.

[15] Monleau M., Bonnel S., Gostan T., Blanchard D., Courgnaud V. (2014). Comparison of different extraction techniques to profile microRNAs from human sera and peripheral blood mononuclear cells. BMC Genomics.

[16] Li Y., Kowdley K.V. (2012). Method for microRNA isolation from clinical serum samples. Anal. Biochem..

[17] Baker M. (2010). MicroRNA profiling: Separating signal from noise. Nat. Publ. Group.

[18] Benes V., Castoldi M. (2010). Expression profiling of microRNA using real-time quantitative PCR, how to use it and what is available. Methods.

[19] Stahlberg A., Kubista M., Pfaffl M. (2004). Comparison of reverse transcriptases in gene expression analysis. Clin. Chem..

[20] Vandesompele J., de Preter K., Pattyn F., Poppe B., van Roy N., de Paepe A., Speleman F. (2002). Accurate normalization of real-time quantitative RT-PCR data by geometric averaging of multiple internal control genes. Genome Biol..

[21] Chugh P., Dittmer D.P. (2012). Potential pitfalls in microRNA profiling. Wiley Interdiscip. Rev. RNA.

[22] Pritchard C.C., Kroh E., Wood B., Arroyo J.D., Dougherty K.J., Miyaji M.M., Tait J.F., Tewari M. (2012). Blood cell origin of circulating microRNAs: A cautionary note for cancer biomarker studies. Cancer Prev. Res..

[23] McDonald J.S., Milosevic D., Reddi H.V., Grebe S.K., Algeciras-Schimnich A. (2011). Analysis of circulating microRNA: Preanalytical and Analytical Challenges. Clin. Chem..

[24] Song J., Bai Z., Han W., Zhang J., Meng H., Bi J., Ma X., Han S., Zhang Z. (2012). Identification of suitable reference genes for qPCR analysis of serum microRNA in gastric cancer patients. Dig. Dis. Sci..

[25] Day E., Dear P.H., McCaughan F. (2013). Digital PCR strategies in the development and analysis of molecular biomarkers for personalized medicine. Methods.

[26] Hindson C.M., Chevillet J.R., Briggs H.A., Gallichotte E.N., Ruf I.K., Hindson B.J., Vessella R.L., Tewari M. (2013). Absolute quantification by droplet digital PCR* versus* analog real-time PCR. Nat. Methods.

[27] Locker G.Y., Hamilton S., Harris J., Jessup J.M., Kemeny N., Macdonald J.S., Somerfield M.R., Hayes D.F., Bast R.C. (2006). ASCO 2006 update of recommendations for the use of tumor markers in gastrointestinal cancer. J. Clin. Oncol..

[28] Lawrie C.H., Gal S., Dunlop H.M., Pushkaran B., Liggins A.P., Pulford K., Banham A.H., Pezzella F., Boultwood J., Wainscoat J.S. (2008). Detection of elevated levels of tumour-associated microRNAs in serum of patients with diffuse large b-cell lymphoma. Br. J. Haematol..

[29] Mitchell P.S., Parkin R.K., Kroh E.M., Fritz B.R., Wyman S.K., Pogosova-Agadjanyan E.L., Peterson A., Noteboom J., O’Briant K.C., Allen A. (2008). Circulating microRNAs as stable blood-based markers for cancer detection. Proc. Natl. Acad. Sci. USA.

[30] Arroyo J.D., Chevillet J.R., Kroh E.M., Ruf I.K., Pritchard C.C., Gibson D.F., Mitchell P.S., Bennett C.F., Pogosova-Agadjanyan E.L., Stirewalt D.L. (2011). Argonaute2 complexes carry a population of circulating microRNAs independent of vesicles in human plasma. Proc. Natl. Acad. Sci. USA.

[31] Turchinovich A., Weiz L., Langheinz A., Burwinkel B. (2011). Characterization of extracellular circulating microRNA. Nucleic Acids Res..

[32] Vickers K.C., Remaley A.T. (2012). Lipid-based carriers of microRNAs and intercellular communication. Curr. Opin. Lipidol..

[33] Valadi H., Ekström K., Bossios A., Sjöstrand M., Lee J.J., Lötvall J.O. (2007). Exosome-mediated transfer of mRNAs and microRNAs is a novel mechanism of genetic exchange between cells. Nature.

[34] Tsuchida A., Ohno S., Wu W., Borjigin N., Fujita K., Aoki T., Ueda S., Takanashi M., Kuroda M. (2011). miR-92 is a key oncogenic component of the miR-17-92 cluster in colon cancer. Cancer Sci..

[35] Diosdado B., van de Wiel M.A., Terhaar Sive Droste J.S., Mongera S., Postma C., Meijerink W.J.H.J., Carvalho B., Meijer G.A. (2009). MiR-17-92 cluster is associated with 13q gain and c-myc expression during colorectal adenoma to adenocarcinoma progression. Br. J. Cancer.

[36] Ng E.K.O., Chong W.W.S., Jin H., Lam E.K.Y., Shin V.Y., Yu J., Poon T.C.W., Ng S.S.M., Sung J.J.Y. (2009). Differential expression of microRNAs in plasma of patients with colorectal cancer: A potential marker for colorectal cancer screening. Gut.

[37] Huang Z., Huang D., Ni S., Peng Z., Sheng W., Du X. (2010). Plasma microRNAs are promising novel biomarkers for early detection of colorectal cancer. Int. J. Cancer.

[38] Schee K., Boye K., Abrahamsen T.W., Fodstad Ø., Flatmark K. (2012). Clinical relevance of microRNA miR-21, miR-31, miR-92a, miR-101, miR-106a and miR-145 in colorectal cancer. BMC Cancer.

[39] Shigoka M., Tsuchida A., Matsudo T., Nagakawa Y., Saito H., Suzuki Y., Aoki T., Murakami Y., Toyoda H., Kumada T. (2010). Deregulation of miR-92a expression is implicated in hepatocellular carcinoma development. Pathol. Int..

[40] Si H., Sun X., Chen Y., Cao Y., Chen S., Wang H., Hu C. (2013). Circulating microRNA-92a and microRNA-21 as novel minimally invasive biomarkers for primary breast cancer. J. Cancer Res. Clin. Oncol..

[41] Jiang Y., Wang H., Cao H., Wang C., Zhang L., Wang H., Liu L., Li Y., Cai J. (2014). Peripheral blood miRNAs as a biomarker for chronic cardiovascular diseases. Sci. Rep..

[42] Asangani I.A., Rasheed S.A., Nikolova D.A., Leupold J.H., Colburn N.H., Post S., Allgayer H. (2008). MicroRNA-21 (miR-21) post-transcriptionally downregulates tumor suppressor Pdcd4 and stimulates invasion, intravasation and metastasis in colorectal cancer. Oncogene.

[43] Kanaan Z., Rai S.N., Eichenberger M.R., Roberts H., Keskey B., Pan J., Galandiuk S. (2012). Plasma miR-21: A potential diagnostic marker of colorectal cancer. Ann. Surg..

[44] Luo X., Stock C., Burwinkel B., Brenner H. (2013). Identification and evaluation of plasma microRNAs for early detection of colorectal cancer. PLoS One.

[45] Köberle V., Pleli T., Schmithals C., Augusto Alonso E., Haupenthal J., Bönig H., Peveling-Oberhag J., Biondi R.M., Zeuzem S., Kronenberger B. (2013). Differential stability of cell-free circulating microRNAs: Implications for their utilization as biomarkers. PLoS One.

[46] Kanaan Z., Roberts H., Eichenberger M.R., Billeter A., Ocheretner G., Pan J., Rai S.N., Jorden J., Williford A., Galandiuk S. (2013). A plasma microRNA panel for detection of colorectal adenomas: A step toward more precise screening for colorectal cancer. Ann. Surgery.

[47] Giráldez M.D., Lozano J.J., Ramírez G., Hijona E., Bujanda L., Castells A., Gironella M. (2013). Circulating microRNAs as biomarkers of colorectal cancer: Results from a genome-wide profiling and validation study. Clin. Gastroenterol. Hepatol..

[48] Wang J., Huang S., Zhao M., Yang M., Zhong J., Gu Y., Peng H., Che Y., Huang C. (2014). Identification of a circulating microRNA signature for colorectal cancer detection. PLoS One.

[49] Morikawa T., Kato J., Yamaji Y., Wada R., Mitsushima T., Shiratori Y. (2005). A Comparison of the immunochemical fecal occult blood test and total colonoscopy in the asymptomatic population. Gastroenterology.

[50] Ahmed F.E., Jeffries C.D., Vos P.W., Flake G., Nuovo G.J., Sinar D.R., Naziri W., Marcuard S.P. (2009). Diagnostic microRNA markers for screening sporadic human colon cancer and active ulcerative colitis in stool and tissue. Cancer Genomics Proteomics.

[51] Ahlquist D.A. (2010). Molecular detection of colorectal neoplasia. Gastroenterology.

[52] Fearon E.R., Vogelstein B. (1990). A genetic model for colorectal tumorigenesis. Cell.

[53] Slaby O., Svoboda M., Michalek J., Vyzula R. (2009). MicroRNAs in colorectal cancer: Translation of molecular biology into clinical application. Mol. Cancer.

[54] Kuo Y.B., Chan E.C., Chen J.S., Shieh F.K. (2013). Fecal miRNAS as biomarkers for the detection of colorectal cancer. J. Gastrointest. Dig. Syst..

[55] Wu C.W., Ng S.C., Dong Y., Tian L., Ng S.S.M., Leung W.W., Law W.T., Yau T.O., Chan F.K.L., Sung J.J.Y. (2014). Identification of microRNA-135b in stool as a potential noninvasive biomarker for colorectal cancer and adenoma. Clin. Cancer Res..

[56] Yau T.O., Wu C.W., Dong Y., Tang C.-M., Ng S.S.M., Chan F.K.L., Sung J.J.Y., Yu J. (2014). microRNA-221 and microRNA-18a identification in stool as potential biomarkers for the non-invasive diagnosis of colorectal carcinoma. Br. J. Cancer.

[57] Wu C.W., Ng S.S.M., Dong Y.J., Ng S.C., Leung W.W., Lee C.W., Wong Y.N., Chan F.K.L., Yu J., Sung J.J.Y. (2012). Detection of miR-92a and miR-21 in stool samples as potential screening biomarkers for colorectal cancer and polyps. Gut.

[58] Koga Y., Yasunaga M., Takahashi A., Kuroda J., Moriya Y., Akasu T., Fujita S., Yamamoto S., Baba H., Matsumura Y. (2010). MicroRNA expression profiling of exfoliated colonocytes isolated from feces for colorectal cancer screening. Cancer Prev. Res..

[59] Ahmed F.E., Ahmed N.C., Vos P.W., Bonnerup C., Atkins J.N., Casey M., Nuovo G.J., Naziri W., Wiley J.E., Mota H. (2013). Diagnostic microRNA markers to screen for sporadic human colon cancer in stool: I. Proof of Principle. Cancer Genomics Proteomics.

